# Mesangial Cell-Specific Antibodies Are Central to the Pathogenesis of Lupus Nephritis

**DOI:** 10.1155/2012/579670

**Published:** 2011-11-28

**Authors:** Guillaume Seret, Yannick Le Meur, Yves Renaudineau, Pierre Youinou

**Affiliations:** ^1^EA2216 “Immunology & Pathology” and IFR146 “ScInBios,” European University of Brittany, 29200 Brest, France; ^2^Unit of Nephrology, Brest University Medical School Hospital, 29609 Brest, France; ^3^Laboratory of Immunology, Brest University Medical School Hospital, BP824, 29609 Brest, France

## Abstract

Not only is nephritis a common complaint in systemic lupus erythematosus, but it is also the most life-threatening complication of the disease. Anti-double-stranded DNA antibodies (Abs), which are found in up to 80% of these patients, might be nephritogenic per se. That is, they may cross-react with mesangial cell (MC) surface proteins, such as alpha-actinin and annexin A2, they may cross-react with mesangial matrix protein such as laminine and fibronectin, or they may recognize chromatin material previously deposited in the glomeruli. The consequence of the binding of anti-MC Abs may be their internalization, which results in activation and proliferation of these MCs. In turn, these activated MCs are suspected of promoting immune complex formation by sequestering and thereby protecting chromatin from degradation. The present paper will explain the mechanisms through which such autoAbs may initiate nephritis.

## 1. Introduction

Systemic lupus erythematosus (SLE) is a nonorgan-specific autoimmune disease, the hallmark of which is a vast array of antiself antibodies (autoAbs), and, among them, the whole range of antinuclear Abs (ANAs). The ensuing immune complexes (ICs) settle in the tissues and thereby subsequently contribute to local damage.

Most organs are at risk of being involved in this process at one time or another, given that the course of the disease consists of sequential flares and remissions. Estimates of the prevalence vary from 20 to 150 cases per 100,000 individuals, with the highest frequency in Afro-Caribbeans, followed by Asians, and far less frequent in Caucasians [1]. The male-to-female ratio rises to 1 : 9 during child-bearing age but diminishes thereafter.

In fact, the pathophysiology of SLE is so complicated that its development implicates multiple genes and entails a number of environmental factors (recognized or unknown). With regard to the genetics, predisposing genes are associated with the innate as well as the acquired immune responses. Of these, SLE can involve the antigen- (Ag-) presenting DR2 and DR3 HLA class II molecules, the lymphocyte activation markers, components of the classical complement activation pathway, various features involved in the processing of ICs, and interferon (IFN) signaling cascade members [2].

Lupus nephritis (LN) predominates as a cause of mortality in SLE and displays several epidemiological particularities [3]. For example, there exists an ethnic susceptibility, in that it develops in 20% of Caucasian patients compared with 50% of Asian patients. Whereas SLE is, by and large, more frequent in females than in males, the susceptibility for LN in Caucasians reaches 50–60% in males compared with 20–35% in females. This complication arises usually within the first two years of the disease. Several gene polymorphisms have been claimed to favor LN (Table 1), and some SLE-specific autoAbs have been shown to recognize glomerular Ags (Table 2). Furthermore, it has been suggested that anti-double-stranded DNA (anti-dsDNA) Ab-induced renal failure could be linked to differences in the fine specificities of these autoAbs. Over several decades, a large body of work has been devoted to deciphering the anti-dsDNA Abs and to understand the deposition of anti-dsDNA/nucleosome ICs in the kidney, yet there are few reports available on the recognition of glomerular structures, and even fewer studies on the recognition of mesangial cells (MCs). Our paper will, therefore, endeavour to provide glimpses into the mechanisms that may account for the development of nephritis in patients with SLE.

## 2. Mesangial Cells

### 2.1. Mesangial Cell Functions

**Figure 1:**
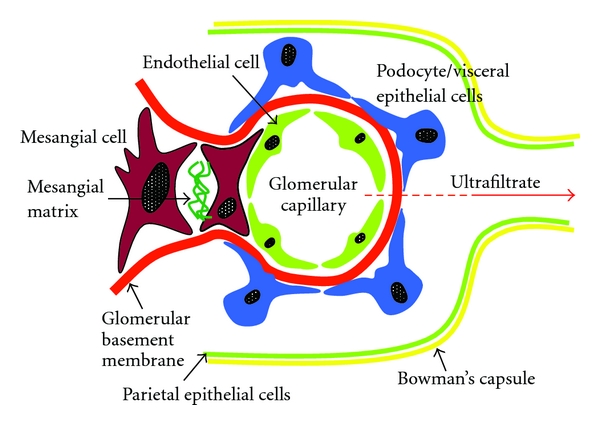
Schematic representation of the different cell types of the glomerular filtration barrier.

Glomeruli are comprised of at least four cell types: MCs, endothelial cells (ECs), and podocytes plus parietal cells, both of an epithelial nature and the later shaping the Bowman's capsule (Figure 1). Filtration through the glomerular barrier is under the control of MCs plus podocytes, along with renal blood flow by contracting the GBM [13]. The glomerular blood-urine barrier superimposes three layers: fenestrae between adjacent ECs, the glomerular basal membrane (GBM), and the slit diaphragm mid podocytes.

 The MCs are specialized smooth muscle cells, of which the contractility depends upon vasoactive molecules, such as angiotensin II and endothelin-1. They possess additional capabilities, including support of the glomerular capillaries. MCs synthesize and renew their own extracellular matrix, which is distinct from the GBM. The mesangial matrix is made up of fibronectin, collagen II, collagen IV, laminin, entactin, nidogen, and perlecan. The sialoglycoprotein fibronectin is located on the MC surface and is required for attachment of circulating components, including chromatin, to MCs and ECs. The other sialoglycoprotein laminin and the sulphated glycoprotein entactin are equally implicated in this event. Other important functions for MCs are their phagocytic capacity to take up apoptotic cells and their capacity to prevent accumulation of ICs by bringing into play nonspecific mechanisms, such as pinocytosis and phagocytosis, and specific mechanisms, such as receptor-dependent processes. Once activated, MCs secrete pro-inflammatory cytokines (e.g.,  interleukin (IL)-1, IL-6, IL-12, and IFN-*γ*), growth factors (e.g., transforming growth factor (TGF)-*β* and vascular endothelial growth factor), and metalloproteinases (e.g., metalloproteinase (MMP)-2 and MMP-9). All these effects are tightly regulated in normal cells and may be markedly altered by glomerular pathology.

### 2.2. Mesangial Cells and Kidney Diseases

A variety of ICs, which are lacking in normal mesangium, become detectable in the kidneys of patients with a variety of diseases, such as LN, IgA nephropathy (IgAN), C1q nephropathy, and mild postinfectious glomerulonephritis (GN). Such patients often present with hematuria, associated with proteinuria at the nephrotic syndrome stage. Much uncertainty surrounds abnormalities of MCs in ICs deposition. Several mechanisms are, in fact, supposed to prevent ICs access into the mesangium. They include the endothelial barrier itself, the effect of a protective glycocalyx, and the recycling capacity of the podocytes that express the neonatal receptor for IgG (FcRn) [14]. The immunoglobulin-specific MC receptor remains a matter of debate, given that the mesangial Fc-gamma receptors are dispensable for kidney injury as well as for cellular activation [15]. Alternatively, nonconventional receptors have been proposed. On the front line of the pathophysiology of IgAN is the transferring receptor, referred to as CD71 [16–18]. The IgA-IgG/CD71 complexes are crucial [19], as suggested by the fact that blocking CD71 with a related monoclonal Ab (mAb) inhibits MC proliferation and cytokine production, namely, IL-6 and TGF-*β*. In addition, IgA and IgG are associated with the complement fraction C3 [20] and the mannose-binding lectin in the mesangium, highlighting the relevance of the complement lectin pathway to the development of such GNs.

Of note, proliferation of MCs and expansion of the mesangial matrix may take place in the absence of ICs. In this context, different forms of glomerular damage develop, namely, diabetic and amyloid nephropathies. In the course of diabetic nephropathy, elevated plasma levels of glucose contribute to the induction of nitric oxide synthase (iNOS), which in turn activate protein kinase C (PKC), mitogen-activated protein kinases (MAPK), and phosphatidyl inosytol-3 kinase/Akt [21]. As a result, fibronectin, collagen IV, and TGF-*β* synthesis are upregulated, leading to the development of fibrosis and resulting in end-stage renal failure. Actually, such is the usual outcome of a large number of GNs.

## 3. Mesangial Cells and Lupus Nephritis

### 3.1. Pathogenic Models

Anti-dsDNA Abs are relevant to the diagnosis of SLE and instrumental in the development of LN. However, the mechanism by which they contribute to the GN is far from clear, considering the fact that not all Abs to dsDNA are able to cause tissue damage to a similar extent. A popular view has been that GN results from ICs associating with nucleosomes released from apoptotic/necrotic cells that have bound anti-dsDNA/chromatin Abs. A wealth of evidence supports this simplistic model. For example, renal flares are preceded by a rise of the anti-dsDNA Ab levels in plasma and a reciprocal reduction in levels of free DNA [22]. In LN, the anti-dsDNA Ab/chromatin complexes are seen as electron-dense structures in the mesangial matrix and move to the GBM as soon as the disease is established [23]. An acquired renal DNase1 deficiency, coupled with chromatin sequestration by matrix protein accumulation, amplifies the process by offering more target Ags to anti-dsDNA Abs [24]. Nonetheless, this mechanism cannot be responsible for the whole process, since analysis of kidney-eluted IgG has revealed that those Abs binding to dsDNA represent as little as 10% of the total bulk of IgG [25]. Additional points to keep in mind are that only a minute fraction of anti-dsDNA Abs are pathogenic when transferred to experimental animals, and LN could develop in the absence of anti-dsDNA Ab. Last but not least, differences between nephritogenic and nonnephritogenic anti-dsDNA Abs are unrelated to structural differences in class, subclass, or avidity (Table 3). Rather, they consist of varying capacities to react with MC products in the absence of a DNA docking site [26]. The generation of nephritogenic Ab is incompletely understood and possibly results from an antigen-dependent stepwise process due to isotype switching and somatic mutations that would result in acquisition of cross-reactivity and high-affinity binding. Stimulation may be sustained by dsDNA along with a glomerular antigen or more probably shared epitopes. It is striking to observe that only one mutation can change the affinity, the cross-reactivity properties, and the kidney binding localization of a pathogenic anti-dsDNA Ab.

Accordingly, the concept has been put forward that anti-dsDNA Abs launch the GN process through cross-reaction with cell-surface and matrix components. So far, several glomerular Ags have indeed been suspected as serving as targets for anti-dsDNA Abs [11, 12]. To reconcile the theory of active cross-reactivity and the concept of passive IC deposition, we reasoned that neither is exclusive and speculated that both are ordered, in that Ab glomerular recognition precedes anti-dsDNA Ab/chromatin deposition [27, 30].

### 3.2. Histology

To account for so much variation in the clinical and histological patterns, the LN histopathological abnormalities have been classified into six classes. Based on the criteria proposed by the International Society of Nephrology/Renal Pathology Society (ISN/RPS) in 2003 [31], they include the morphology of the lesions, their mesangial, endothelial and epithelial extent, the Ab deposition, and the distinction between active and chronic lesions. Briefly, class I histopathological damage corresponds to mesangial deposits, but renal symptoms may be absent. Class II refers to mesangial proliferation, and mild proteinuria and microscopic hematuria characterize these patients. The renal prognostic value is often excellent but may evolve through mesangial and endothelial lesions [32, 33]. Class III and class IV imply glomerulus antibody deposition. In essence, class III LN (less than 50% of the glomeruli are impacted) manifest hematuria, proteinuria, nephritic syndrome, and occasionally hypertension. Class IV (more than 50% of the glomeruli) characterizes diffuse LN and comprises segmental and global forms, according to the severity of glomerular lesions. Hematuria, massive proteinuria, nephritic syndrome, and acute renal failure occur in 16% of class IV patients. Class V corresponds to immune-complex-derived membranous nephritis. The lesions display global or segmental distribution, although more than 50% of the capillary basement membrane is involved in either case. Clinical presentations include proteinuria (typically at a nephritic range), with hematuria but usually without renal insufficiency. Finally, class VI lesions correspond to the last stage of the disease, resulting from the alteration between flares and pauses, leading to overt renal failure, and substantiated by vascular sclerosis, tubulointerstitial scarring, and glomerular sclerosis. However, these clinical features are not well associated with the classification since, histologically, severe LN may be clinically silent. Besides these well-documented types of damage, SLE yields a broad variety of vascular lesions, which are neglected in the ISN/RPS 2003 classification.

### 3.3. Mesangial Cells in Lupus Nephritis

Aberrant proliferation, apoptosis, and activation of MCs are common findings during LN. As a consequence, numerous genes have been demonstrated by immunohistochemistry and/or molecular biology to be upregulated during LN [34–38]. These include genes for survival and apoptotic factors (Bcl-2, Fas, FasL), chemokines that attract inflammatory cells (CCL5, CXCL1), inflammatory mediators (ROS, iNOS), proinflammatory type 1 cytokines (IFN-*γ*, IL-12, IL-6), mesangial matrix synthesis (fibronectin), collagen IV degradation (MMP-2 and MMP-9), and chromatin accumulation (DNase1 down-regulation). MC pathogenicity could be attributed in part to anti-dsDNA activity since anti-dsDNA Abs stimulate MCs to produce chemokines (MCP-1, CCL-5), matrix metalloproteinases (MMP-2, MMP-9), reactive oxygen (iNOS), cytokines (IL-6, TGF-*β*), and lipocalin-2/NGAL [39, 40]. Although incompletely characterized, such effects are related in part to the activation of the PKC and MAPK pathways.

## 4. Autoantibodies and Lupus Nephritis

### 4.1. Antiglomerular Antibodies

ANAs may arise well before the development of overt disease, with a crescendo of more and more SLE-specific autoAbs being produced over 10 years [41]. The earliest ANAs are anti-Ro/Sicca Syndrome (SS)-A and anti-La/SSB Abs, on average 3.7 years before, followed by anti-dsDNA Abs, on average 2.2 years before, and the anti-Smith (Sm) ribonucleoprotein (RNP) Abs, on average 0.9 years before the advent of clinical symptoms. Intriguingly, the presence of anti-Ro/La/Sm RNP Abs and IgM anti-*β*2 glycoprotein I could well protect the patient from LN [42, 43]. On the other hand, high-titer and high-avidity anti-dsDNA Abs have been reported to be linked to active disease and suspected to be associated with LN.

Typically, ICs from patients suffering LN contain IgG, IgM, and IgA, along with the complement fractions C1q and C3. In 90% of the cases, IgG predominates over IgM and IgA which are associated with 60% of the IgG-containing ICs. These latter abnormalities are exceptional in diseases other than LN. With regard to fibrin and fibrinogen, they characterize crescent and necrotizing segments. Specificity analysis of Abs eluted from the kidneys unveils a broad range of reactivities. These are chromatin, *α*-actinin, collagen, entactin, fibrinogen, laminin, proteoglycan, phospholipids (PLs), myosin, RNP, and so on [25]. Similarly, microarray technology has distinguished two main clusters of serum IgM and IgG autoAbs in the serum of patients with LN, based on their specificities. One is directed to chromatin and the other to the glomerulus [44]. Their DNA dependence has been tested using DNase-1 pretreatment, and the results of these experiments indicated that 20% of the Abs binding to the glomeruli were DNA independent.

The observation that some anti-dsDNA Abs attach directly to renal tissues, and more particularly to MCs, raises the question as to whether or not any target Ag is specific for such LN-associated autoAbs. This issue has been addressed using several approaches. First, anti-dsDNA mAbs have been injected into nonautoimmune mice and shown to cause a LN-like disease [26, 45]. Similarly, immunization with a peptide for anti-dsDNA Ab can initiate LN in Balb/c mice [46]. Of note, site-directed mutagenesis of the nephritogenic anti-dsDNA mAb R4A alters not only its affinity to dsDNA, but also its cross-reactivity with glomerular Ags. Cross-reactivity can even shift from the glomerular to the tubular area [28]. Anti-dsDNA Ab point mutations may thus influence the evolution of LN over time. The second approach relied on glomerular-derived peptides which were examined for their interactions with anti-dsDNA Abs [47]. The third approach used human sera purified from LN patients and those which recognized human MCs as well [48]. This approach enabled the discovery of three main specific MC targets at 42, 63, and 74 kDa when using anti-dsDNA and non-anti-dsDNA purified Abs from these patients. DNase1 pretreatment did not affect their binding. Furthermore, purified antihuman MC Abs are likely to be internalized and thus able to encourage iNOS activation, MC proliferation, and matrix synthesis [12]. As recently documented [49], antihuman MC Abs are associated with 84% of active LN compared with 43% of inactive LN.

### 4.2. Antimesangial Cells Antibodies

#### 4.2.1. Anti-*α*-Actinin Antibodies

Glomerular *α*-actinin is expressed on the surface of MCs and podocytes but not on that of the GBM. This actin-binding protein belongs to the superfamily of cytoskeletal proteins. It is comprised of four isoforms, and mutations in the fourth isoform can lead to focal and segmental glomerulosclerosis [50].

That *α*-actinin can be targeted by anti-dsDNA Abs has also been demonstrated. This is tied to the fact that injection of anti-dsDNA mAb into RAG-1-deficient mice induces a proteinuria with glomerular deposits in these animals. Cross-reaction with *α*-actinin [26] or laminin-1 [51] provided the anti-dsDNA Abs with the capacity to impair the renal function. This view was supported by the finding that, once bound to MCs, anti-dsDNA R4A mAbs [29] upregulate the production of iNOS and proinflammatory chemokines [39]. Demonstration of the reality of pathogenic *α*-actinin, which is worthy of pursuit in the future, was thus reinforced by the observation that *α*-actinin-immunized normal mice mounted an anti-*α*-actinin Ab response first and then produced anti-*α*-actinin and anti-chromatin Abs, along with advancing stages of the LN-like disease [52]. In SLE patients, the anti-*α*-actinin Ab production culminates early at the initiation of the LN, but their titers drop dramatically after treatment is initiated, that is, when the disease activity is reduced. We must admit that, in contrast to the anti-dsDNA/chromatin activity, the results of the detection of these autoAbs are inconsistent [53–56]. Of interesting note, the anti-*α*-actinin response is related to the actin-binding site of *α*-actinin [54, 57].

#### 4.2.2. Antiannexin A2 Antibodies

Annexin A2 is a calcium-dependent PL-binding protein expressed on the surface of phagocytic cells, such as macrophages, ECs, and MCs. This protein is pivotal in the regulation of MC proliferation, activation, apoptosis, and in coagulation by recruiting plasminogen and tissue plasminogen activator.

In LN, IgG, and C3, deposits colocalize with annexin A2 in the glomeruli but, surprisingly, not in the tubuli [12]. Annexin A2-dependence has been tested by gene silencing using RNA interference technology, as an attempt to establish that its downregulation prevents anti-dsDNA Ab binding, Ab internalization, and MC activation. Supporting this view, a positive antiannexin A2 Ab test is associated with active LN and thrombosis [12, 58]. The abnormality is related to the activation of the tissue factor on ECs and monocytes, which is in accord with the detection of anti-annexin A2 Ab in 40% of patients with the anti-PL syndrome.

### 4.3. Antimatrix Antibodies

#### 4.3.1. Antilaminin Antibodies

Laminin belongs to the mesangial matrix. Laminin-1, which is the most abundant isoform, is derived from MCs. It is overexpressed and hence becomes detectable in the GBM during LN and at the periphery of end-stage sclerotic lesions [59]. It is, therefore, of no surprise that antilaminin Abs are found during LN and that their levels correlate with the disease activity and proteinuria [51]. Notwithstanding, they are not specific for SLE, being also detected in recurrent miscarriages, infertility and pemphigus. The main epitope recognized by antilaminin-1 Ab corresponds to the binding site of laminin to the basement membrane receptors.

#### 4.3.2. Antifibronectin Antibodies

Fibronectin is absent from normal mesangial matrix but overexpressed in LN and colocalized with IgG/chromatin ICs in the mesangium. The prevalence of antifibronectin Abs ranges from 30 to 80% in patients with SLE, and from 15 to 40% in those with rheumatoid arthritis and other systemic vasculitis. Although nonspecific for any disease, antifibronectin Ab levels correlate with activity in patients with SLE. In this regard, one of the most efficient drugs to treat LN, mycophenolate mofetil, prevents anti-dsDNA Ab-induced fibronectin production by MCs. In other words, the drug contributes to reduce IC deposition [40].

### 4.4. Miscellaneous Antibodies

Involved in the elongation step of protein synthesis when associated with the large ribosomal subunit, the ribosomal serine phosphorylated proteins P0, P1, and P2 appear on the membrane of multiple cells, including MCs and blood cells of patients with SLE. The reported prevalence of antiribosomal Abs varies from 5 to 45% in SLE, more often in Asian patients than in Caucasian and African patients [60]. In SLE, they are restricted to active disease, with kidney, hepatic, and neuropsychiatric complications. Once again, high-affinity anti-dsDNA Abs cross-react with ribosomal proteins.

Alpha-enolase appears on the surface of MCs and podocytes and in the tubuli from patients with LN. In this setting, it acts as a glycolytic enzyme and a receptor for plasminogen. The anti-*α*-enolase Ab test is positive in SLE patients but is not associated with LN and flares [61]. Alpha-enolase has been identified as an autoAg in other diseases, such as Behcet's disease, retinopathy, and severe asthma.

## 5. Conclusion

**Figure 2:**
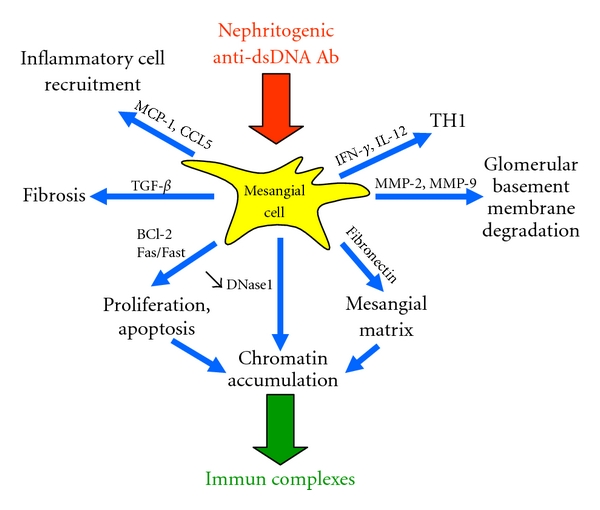
A working model of mesangial cell (MC) stimulation by nephritogenic anti-dsDNA Ab in lupus nephritis leading to accumulation of immune complexes.

Whereas compelling evidence in LN suggests a pathogenic role for anti-dsDNA Abs, their detailed mechanisms of action are not restricted to IC formation. As illustrated in Figure 2, we propose that, among anti-dsDNA Abs, a minute fraction of anti-dsDNA Abs stimulate MCs to produce cytokines, chemokines, and matrix metalloproteinases important in the initiation of the inflammatory process. In addition, such activation is associated with proliferation and apoptosis, matrix protein accumulation, and a reduction of DNase1 activity that would, in turn, contribute to the formation of anti-dsDNA Ab chromatin/ICs in the mesangium and later in the GBM that characterize severe LN. Furthermore, while the focus of this paper is on MCs, it should be mentioned that antigens could be displayed by other glomerular cells as well, including podocytes. As a consequence, the pathogenicity of these Abs would be enhanced by targeting more than one cell type. 

## Figures and Tables

**Table 1:** Genes associated with lupus nephritis (LN) [4–10].

Gene	Function	Influence
CD48	Leucocyte adhesion	Protective effect
Fc*γ*RIIIA/IIA	Binding affinity	Susceptibility to SLE and LN
Kallikrein	Inflammation	Protective effect
IL-18	Inflammation	Susceptibility to LN
Myeloperoxidase	Inflammation	Susceptibility to LN
TLR9	Immune response	Susceptibility to LN
MBL2	Complement	Susceptibility to LN

**Table 2:** Glomerular targets for anti-double-stranded (ds)-DNA antibodies [11, 12].

Molecules that directly cross-react with anti-dsDNA antibodies	Cell type/glomerular matrix
Alpha-actinin	Mesangial cells
Annexin A2	Mesangial cells, epithelial cells
Ribosomal P protein	Mesangial cells, endothelial cells
Alpha-enolase	Mesangial cells, epithelial cells
Laminin	Glomerular matrix
Fibronectin	Glomerular matrix
Collagen	Glomerular matrix
Heparan sulfate	Glomerular matrix
Hyaluronic acid	Glomerular matrix

**Table 3:** Nephritogenic and cross-reactive anti-dsDNA Ab properties [26–29].

	Non-nephritogenic anti-dsDNA Ab	Nephritogenic anti-dsDNA Ab	Cross-reactive anti-dsDNA Ab
Class	IgG, M and A	IgG mainly	IgG mainly
Somatic mutations	No	Yes	Yes
Affinity	Low	High	High
Cross-reactivity	No	Yes	Yes
Living cell internalization	No	Yes	suspected
Glomerular direct binding	No	Yes	Yes
Proteinuria	No	Yes	mainly
